# An Efficient Edge Computing-Enabled Network for Used Cooking Oil Collection

**DOI:** 10.3390/s24072236

**Published:** 2024-03-31

**Authors:** Bruno Gomes, Christophe Soares, José Manuel Torres, Karim Karmali, Salim Karmali, Rui S. Moreira, Pedro Sobral

**Affiliations:** 1 Faculty of Science and Technology, University Fernando Pessoa, 4249-004 Porto, Portugal; bagomes@ufp.edu.pt (B.G.); csoares@ufp.edu.pt (C.S.); jtorres@ufp.edu.pt (J.M.T.); rmoreira@ufp.edu.pt (R.S.M.); 2 Hardlevel—Renewable Energies, 4410-235 Vila Nova de Gaia, Portugal; karim.karmali@hardlevel.pt (K.K.); salim.karmali@hardlevel.pt (S.K.); 3 LIACC—Artificial Intelligence and Computer Science Laboratory, University of Porto, 4200-465 Porto, Portugal

**Keywords:** edge computing, intelligent edge system, fog computing, delay-tolerant networks, smart city, domestic used cooking oil (UCO) collection, recycling

## Abstract

In Portugal, more than 98% of domestic cooking oil is disposed of improperly every day. This avoids recycling/reconverting into another energy. Is also may become a potential harmful contaminant of soil and water. Driven by the utility of recycled cooking oil, and leveraging the exponential growth of ubiquitous computing approaches, we propose an IoT smart solution for domestic used cooking oil (UCO) collection bins. We call this approach SWAN, which stands for Smart Waste Accumulation Network. It is deployed and evaluated in Portugal. It consists of a countrywide network of collection bin units, available in public areas. Two metrics are considered to evaluate the system’s success: (i) user engagement, and (ii) used cooking oil collection efficiency. The presented system should (i) perform under scenarios of temporary communication network failures, and (ii) be scalable to accommodate an ever-growing number of installed collection units. Thus, we choose a disruptive approach from the traditional cloud computing paradigm. It relies on edge node infrastructure to process, store, and act upon the locally collected data. The communication appears as a delay-tolerant task, i.e., an edge computing solution. We conduct a comparative analysis revealing the benefits of the edge computing enabled collection bin vs. a cloud computing solution. The studied period considers four years of collected data. An exponential increase in the amount of used cooking oil collected is identified, with the developed solution being responsible for surpassing the national collection totals of previous years. During the same period, we also improved the collection process as we were able to more accurately estimate the optimal collection and system’s maintenance intervals.

## 1. Introduction

The goal of IoT was, from the beginning, to create an interconnected network of smart physical objects [1]. After 20 years from its first appearance, the generic definition of IoT is still evolving. From networks to analytics, many knowledge areas have been contributing to the IoT exponential growth.

Even without noticing, our daily lives are already driven by the technological advances in smart connected devices. For instance, by simply visiting a new city, a person can be surrounded by a multitude of IoT solutions/products. The navigation system of his smartphone guides the person to the city’s points of interest, smart occupancy sensors ease the task of finding a free parking slot, and Bluetooth beacons provide contextual information according to the user’s localization, while smart security systems constantly monitor citizens for any kind of suspicious behavior. With the goal of providing services/solutions for citizens in an urban context, smart cities nowadays are one of the prominent domains in the IoT universe [2]. Among solutions like city monitoring systems and security-oriented systems, waste management emerges, in the context of smart cities, as a way to tackle the excessive garbage generated by the world’s population [3]. Although an efficient waste management system is required in order to prevent the harmful consequences of the increasing waste volume [4] some technological challenges are still imposing barriers in the quest for an optimal solution, as discussed in Section 2.

**Figure 1:**
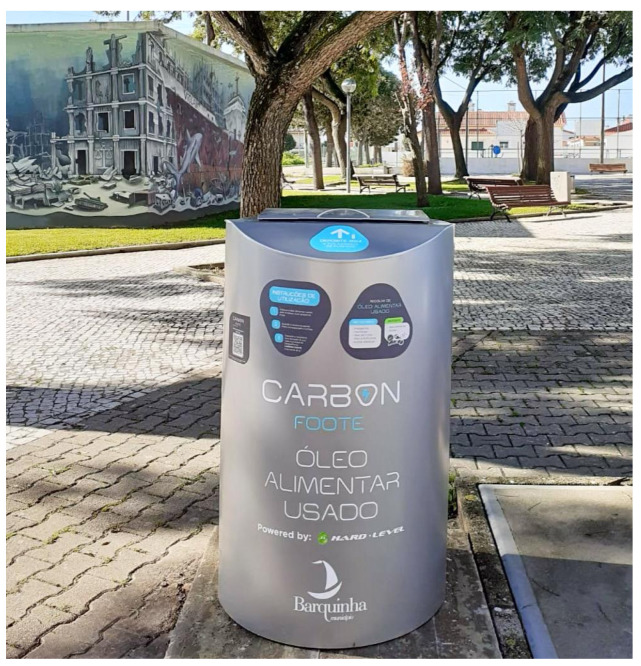
Hardlevel smart oil bin.

With more than fifteen years working as a waste management operator, Hardlevel [5] is nowadays a national (Portugal) reference on used cooking oil (UCO) management. In addition to collection, processing, and storage of UCO from the industrial sector, since 2017, Hardlevel has embraced the challenge of developing and deploying a countrywide network of smart cooking oil collection bins (see Figure 1), considering the interest of three main stakeholders:

And private entities responsible for public areas—Typically, domestic cooking oil collection points, located in public spaces, tend to suffer from the accumulation of dirt and grease caused by spills and excess of deposited waste [6]. Despite this, the need to have appropriate oil disposal points, the collection bins, became essential in the city landscape. Increasing the ratio of the collected oil requires a broad number of deployed collection bins; thus, the product needs to be positioned as a commercially interesting solution attracting public entities and clients with the promise of a clean, city-blended proposal for domestic cooking oil collection.

Citizens/domestic users—Even considering the lack of an understanding of the collection network importance, the percentage of UCO forwarded to recycling is greatly impacted by the number of users that, either by not knowing, or not caring, still dispose of this harmful waste incorrectly. Raising awareness among citizens stands as the first step to increasing the number of active users. In addition to spreading the word on the relevance of UCO recycling, a smart collection bin enables the registration of users and their disposals, allowing for the introduction of loyalty programs to gamify the UCO disposal experience [7].

Waste management operators—Every on-field intervention entails operational and environmental costs that, from the waste management operator standpoint, need to be minimized. A smart cooking oil collection bin allows for a paradigm shift in oil collections performed by the management operator staff. Instead of collecting oil on a prescheduled periodicity, the filling level monitoring detection ensures, for instance, that collections happen only when required, thus contributing to the system efficiency. Additionally, a smart UCO collection bin enables monitoring the trustworthiness of each disposal, consequently reducing the chances of collecting unwanted waste (e.g., water or mineral oil).

Given the obtained results in a study funded by Hardlevel [6] on the state of the UCO recycling in Portugal, two distinct UCO collection success metrics were identified:

User engagement—According to [6], in Portugal, only 1.8% of all the used cooking oil produced by the domestic sector is forwarded for recycling. Given that this residue, when incorrectly disposed of, constitutes an environmental threat, and it may also have the potential to be reused as an alternative energy source, the first goal of the system would be to raise awareness among citizens for the importance of UCO recycling. Based on the disposal detection and counting enabled by a smart bin, gamification techniques, such as campaigns and point programs, should be explored with the aim of increasing the number of active users.

Collection efficiency—In order for the UCO collection process to take place, a widespread network of collection trucks needs to be frequently moving across the country gathering oil from all the collection bins. Filling-level-based collection and preventive maintenance are some goals of an IoT-enabled collection bin, reducing the number of visits and consequently increasing the system efficiency.

It is worthwhile mentioning that the UCO collection bins, installed and operated by Hardlevel until 2018, belonged to a previous product generation, lacking the desirable edge processing capabilities of the current, state-of-the art UCO bins.

The development and deployment of a smart, edge-intelligent, UCO collection network reveals some interesting challenges: the necessity to scale up to thousands of deployed units in heterogeneous installation contexts; the detection of new disposal events, carried out by domestic users, in each UCO collection bin; and the user authentication in the UCO collection net. All these aspects make the cooking oil collection an interesting problem to assess the relevance and suitability of established and emerging computing paradigms of cloud, fog, and edge computing for the IoT, as described in Section 3. In Section 3.1, the most relevant requirements are identified. The SWAN details and layered architecture are presented in Section 3.2. By presenting a solution to the problem of household cooking oil collection, the proposed SWAN system can also have a positive social and environmental impact. The major improvements, made possible by this solution, are presented in Section 3.3. Results from four years of observations are presented in Section 4, evaluations of the system performance on user engagement (see Section 4.1) and collection efficiency (see Section 4.2) metrics, corroborating the relevance of the proposed solution. Finally, Section 5 provides final remarks for this article.

## 2. Computing Paradigms for the Internet of Things

From the extra network load generated by the communication of billions of devices [8], to the limited energy availability [9], the IoT needs to overcome some challenges to emerge as a definitive solution for the waste management problem. In [1], the authors compiled a list of the challenges faced by IoT, from which we highlight the following:

- Energy management—research should be conducted on energy harvesting solutions, as well as minimizing the used energy during operation;
- Scalability—considering the increasing number of active connected devices, this can raise some concerns regarding the scalability of the standard hierarchical cloud computing paradigm for the IoT;
- Security and privacy—given the low availability of resources, most of the time, advanced security techniques are not implemented by IoT devices. The chosen computing architecture may increase the security and protect privacy, limiting access from external unknown entities;
- Communication–one of the main technological challenges facing IoT. The lack of a silver bullet on a trade-off between coverage, data rate, and energy consumption is still impacting communication-dependent solutions.

Although disjoint, the computing paradigm emerges as the common ground between the four challenges. Therefore, a disruptive change from the standard cloud computing to an edge computing approach could potentially contribute to surpassing most of the challenges faced by the IoT. It is defined by the US National Institute of Standards & Technology [10] as a low-management-intensive model for enabling ubiquitous, on-demand access to a shared pool of configurable resources.

Cloud computing becomes a standard on where to store and process data. Given its working principles, cloud computing appears as a service, closing the technological gap between broad and small organizations [11]. The greater simplicity, high availability of computational resources, and centralized nature [12] make the cloud relevant for IoT applications, allowing for the convergence of data generated by a widely spread network of *things*. However, the required link between the field and the cloud layer raises efficiency (energy and time) and security challenges for these resource-constrained devices [1]. To overcome these challenges, while also tackling the problems of mobility and context awareness [13], data processing and storage must occur closer to the sources (the edge). Edge computing gives the end-nodes the possibility to simultaneously consume and produce data, leading to a paradigm-shift on the IoT [14]. By offloading part of the problem to the edge of the network, edge computing inherently provides benefits on aspects like scalability and latency. Since the edge device has the ability to locally process the collected data, sensitive data do not need to be exchanged, therefore eliminating the need for complex (secure) data transmission techniques [12].

In 2015, Cisco introduced the concept of fog computing as an extension to the classic cloud computing [15]. Aiming to surpass the problems of latency and bandwidth aroused with the growing needs of IoT systems, the fog specifies an area close to the data sources, in which a heterogeneous network of devices is left in charge to locally produce and act on field data [15]. With inspiration in IoT applications with strict demands, not easily achieved by the standard cloud computing paradigm, Ref. [16] suggests fog computing as the solution for the problems of scalability, response times, data quality, and localization awareness. Although the understanding definition of fog computing can potentially overlap with the edge one [12], when both layers are combined in a network topology, fog computing may appear as an interface to attach end-nodes (the edge) to the cloud [12].

The three computing paradigms present different benefits and drawbacks. IoT applications should still count on the cloud to centralize and manage the device networks, while the movement of computing and storage to the edge can reduce latency and security/privacy concerns. Table 1 presents a comparison between the three computational paradigms.

### 2.1. From Edge Computing to Edge Intelligence

Artificial intelligence (AI) can significantly ease the task of gathering information from the data collected and generated by IoT devices, giving IoT applications the ability to learn without being explicitly programmed [17]. The movement of AI to the network’s edge in the next two to five years was predicted by Gartner in 2019 [18]. Along with the potential to unleash new possibilities [19], bringing AI to the IoT raises scalability challenges for the cloud computing paradigm. Machine learning (ML) enables devices to improve through experience [17], and can usually be discretized into two tasks: building a model from a broad set of training data (training) and drawing conclusions based on the generated model (inference) [19]. Both tasks are resource-intensive, increasing the relevance of load balancing them according to the benefits and drawbacks of each of the computing layers. In [19], a conceptual overview of edge AI was given. In a standardization effort, an edge intelligence classification chart was laid out, focusing on distinguishing between the different levels of load balancing in the train and inference tasks. Seven levels were specified, from cloud training and inference to the all on-device approach.

In [20], the authors presented an architecture aiming to solve the problems of network latency, data volume, and load balancing. In this approach, as opposed to the standard cloud computing architecture, the resource-intensive task of classifying or detecting objects on a captured frame is accomplished by the edge device. The authors highlighted examples mainly applied to drone operation, where a drone searches for people to rescue on natural disasters, or for specific weeds on which to spray a weedkiller in a smart agriculture context, but no concrete implementation of the abovementioned solutions was evaluated.

Combining the concepts of IoT with the movement of artificial intelligence to the edge of the network brought researchers to establish a new branch on IoT systems—Artificial Intelligence of Things (AIoT). Ref. [21] set the scene on the future of IoT, while exploring the applications and challenges associated with running artificial intelligence tasks on power-constrained devices. Also, considering the hardware point of view, an analysis of the trade-off between power efficiency and programming flexibility for different types of processors was laid out.

An in-depth analysis on the convergence of edge computing and deep learning was presented in [22]. Due to the same heterogeneity referred to in [23], the blurred boundaries of edge computing justify a differentiation between on-device intelligence and edge intelligence. A comparison chart was presented, rating on-device, edge, and cloud intelligence considering six parameters: privacy, latency, diversity, scalability, on-device cost, and reliability.

### 2.2. Related Work on Edge Computing Applied to Smart Waste Management

Technological advances on the Internet of Things are rapidly making their way into smart cities. Given the problem of the excessive waste generated in our societies, many solutions have been studied and deployed with the aim of improving waste management operations. The challenges and opportunities for IoT-enabled waste management were presented in [24]. In addition to an understanding and comparison of the waste management models, the authors highlighted the benefits of using smart devices in order to improve the tasks of waste collection and transport. The proposed IoT-based waste management is classified in three main categories: physical infrastructure, IoT technology, and software analytics. With a focus on the IoT technology part of the problem, a search for related work on waste management was conducted, prioritizing projects directly related to UCO management over generic waste management.

With the aim of optimizing oil collection from restaurants, Ref. [25] presented a solution for improved collection efficiency based on the filling level of used cooking oil containers. Although some considerations were made about the sensing solution (load cells), the main focus was kept on route optimization algorithms.

Moreover, on the subject of used cooking oil collection from restaurants, a power-efficient, edge-computing-enabled solution was proposed in [26,27]. Both works refer to the same project. While [26] focused on the development of a power-efficient, context-aware edge device for liquid level monitoring, in [27] the authors targeted the specification of a scalable fog-computing-based approach considering an application layer gateway local to each restaurant. The proposed topology allows for cost-effective edge devices with low energy consumption values.

The authors in [28] presented a survey on the related work on IoT-based solid waste management solutions. Considering the used comparison criteria, a logical AND was applied in the following: filling level monitoring; publicly available, recycling points; defined computing architecture; support for dynamic schedule and routing of waste collection. Related works with an evaluation based on exclusively synthetic results were not considered. In [29], an IoT platform for smart municipalities solid waste management was proposed. The project described the state of the art for waste collection in the city of Wuhan, with the authors highlighting an expensive collection task (both economical and environmental) and suboptimal environmental awareness as the main issues.

In [30], the authors proposed an edge-computing solution for automated detection of plastic-bag contamination in waste. The system enhances waste collection efficiency by reducing manual labor and increasing the contamination detection speed. It demonstrates scalability through its deployment on waste collection trucks and its ability to perform detection relying on on-site (edge) processing.

Most of the related work on cooking oil collection still relies on standard collection bins, without any route optimization or user engagement techniques. Looking at the more understanding concept of waste management, multiple projects (see Table 2) are already implementing solutions to increase the collection efficiency and even finding ways to encourage citizens to correctly dispose of their waste. Although these projects tend to focus only on one of the optimization problems, most of the time they are evaluated against exclusively synthetic datasets. Leveraging the benefits brought about by each of the computing models, a collaborative cloud–fog–edge architecture has the potential to provide efficient disposal and collection tasks.

## 3. SWAN System Specification

The way that UCO is disposed of has a direct influence on its environmental impact. While treated oil has the potential to be recycled and reused as a renewable energy source, improperly disposed UCO can present a harmful agent to many ecosystems [32]. Knowing that the vast majority of the domestic cooking oil is not forwarded towards recycling [6], an efficient solution for oil collection is needed. This section focuses on the specification of a state-of-the-art, edge-intelligent architecture to cope with the requirements of an efficient, widely spread network of collection bins. The methods and techniques applied in each of the network layers are also discussed.

### 3.1. Requirements

In order to meet the interests of the involved stakeholders, five initial key requirements/restrictions were set for the smart cooking oil collection network:

- Energy availability: every edge device (smart collection bin) must be battery powered, with an emphasis being placed on reducing its energy footprint to a minimum. If possible, energy harvesting solutions, such as solar, could be applied in order to achieve a self sustainable energy source [33].
- Unitary cost: since the solution must rapidly scale and adapt to an ever-growing network of collection units, the unitary cost will always be considered when choosing between approaches and technologies.
- Data security: sensitive data should always be protected when stored, transferred, or processed, in agreement with the data protection rules [34].
- Network connection: some edge units may be installed in remote areas where a scenario with no network coverage must be considered.
- Data quality. The telemetry sent by each of the collection bins determines whether a collection is required. Therefore, fields like filling level or number of disposals should reach the cloud reliably (minimizing missed communications) and accurately (close to the real values).

### 3.2. SWAN Architecture

**Figure 2:**
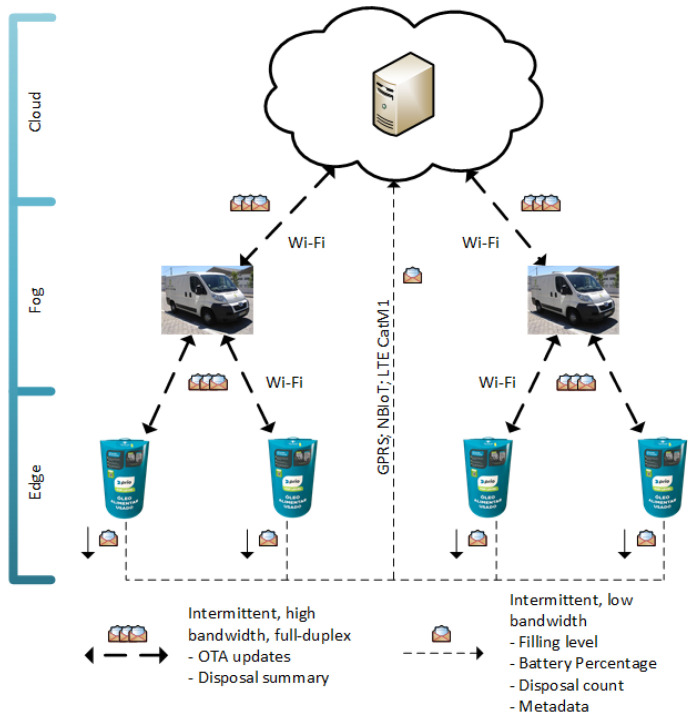
SWAN system architecture.

The previous generation of the UCO collection bin, the standard cloud computing approach, has some clearly identified problems, namely, it represents a suboptimal solution on aspects like service availability, operation costs, or network offloading, with an impact on the domestic user (stakeholder) experience. Conversely, an exclusively edge processing approach to the problem would increase the complexity and effort of managing a widespread and decoupled network of collection units (as mentioned in Section 2). Figure 2 presents the Smart Waste Accumulation Network (SWAN) system architecture, which implements an IoT-based cooking oil collection network.

Taking advantage of the benefits of each of the computing paradigms discussed in Section 2, SWAN converges cloud, fog, and edge layers.

#### 3.2.1. Cloud Layer

By establishing a common ground between users and IoT devices, the cloud layer can potentially ease the system’s management requirements. Given its simplicity, interoperability, and high resource availability [13], SWAN counts on the cloud layer for centralized data storage and execution of computer intensive tasks (see Table 1). This layer generates and maintains all the device’s configuration and firmware versions, with a focus also on the cloud to later train machine learning models for oil classification with edge inference [35].

**Figure 3:**
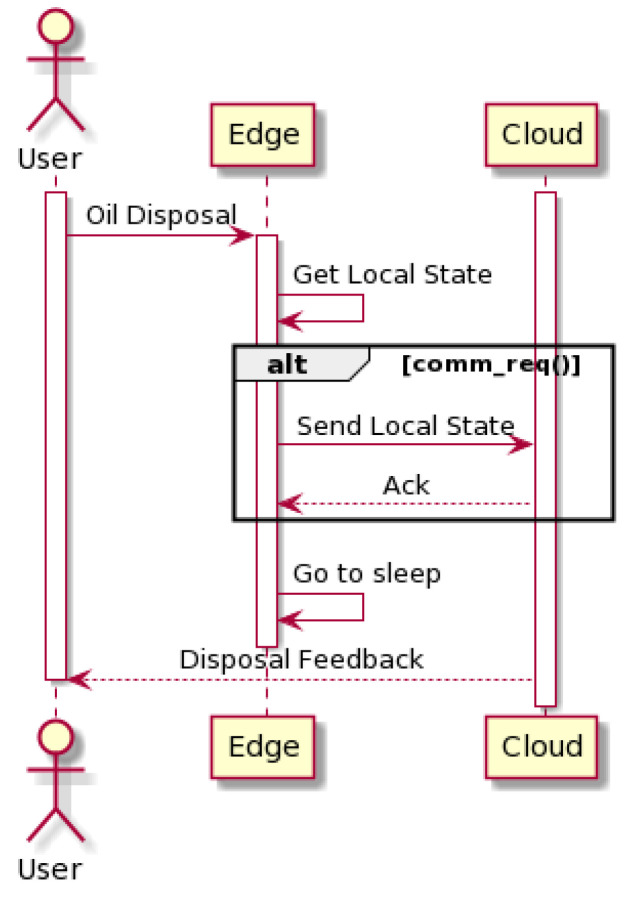
Cloud sequence diagram.

Figure 3 displays the message sequence between the user, edge, and cloud after an oil disposal. The cloud layer should keep track of all the collection bins running parameters and settings (e.g., communication interval, firmware version, communication endpoints, etc.), as well as listening for the arrival of new, field generated, information (e.g., filling level, number of disposals, battery state of charge, etc.). To finish this process, the in-house-developed cloud IoT platform [36] should notify the user with its disposal feedback.

#### 3.2.2. Fog Layer

**Figure 4:**
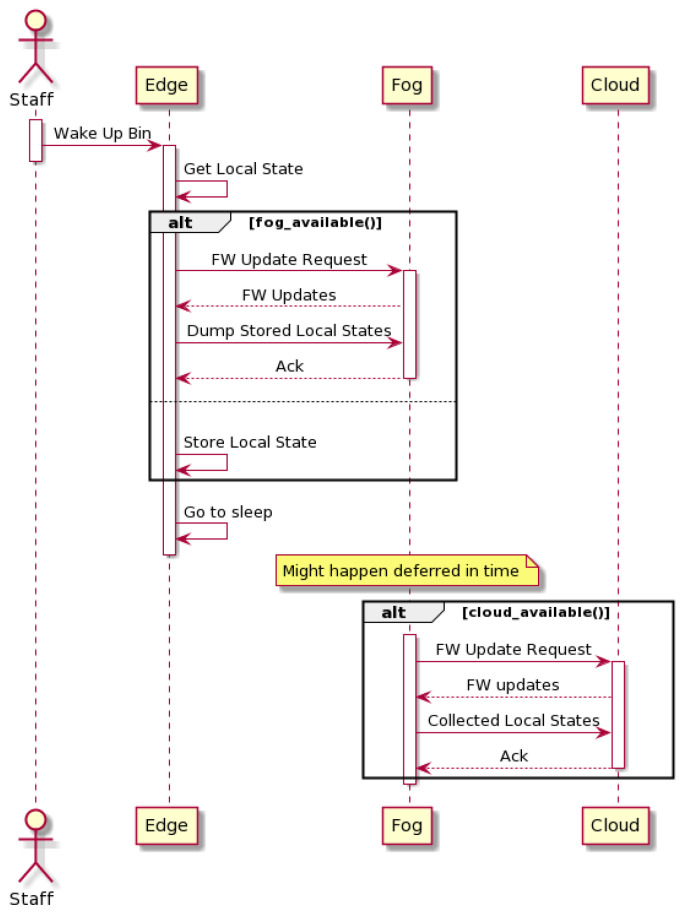
Fog sequence diagram.

Since many collection bins are deployed in environments with weak network coverage, the specified architecture needs to cope with scenarios where resources are not constantly available. This service availability based on partial intermittent resources is made possible by leveraging the company’s collection trucks. With the integration of a microprocessor on each of the collection trucks, a delay-tolerant network is introduced by the fog layer. Figure 4 displays the message exchange after a disposal in a smart bin without any wide area network connection. The vehicular fog layer [37] is responsible for opportunistic (during the visit by a member of the collection team) downstream firmware upgrades and updated trained models. It also delivers, in the cloud, all the information collected from disconnected collection bins, allowing for a deferred convergence.

#### 3.2.3. Edge Layer

SWAN poses itself as an edge computing architecture, leaning the complexity towards the layer materialized by the smart collection bin. Figure 3 and Figure 4 display the smart bin interaction with the remaining participants when depending, respectively, on the cloud or exclusively the vehicular fog. Every time an oil disposal is detected, the smart bin wakes up and performs a series of actions depending on its context. It starts by crossing variables like filling level, number of disposals, and time elapsed since the last cloud connection to assess if a communication is required; then, if a wide area network is available (GPRS, LTE Cat-NB, and LTE-Cat-M1 are currently being used), the edge device pushes to the cloud its current local state (see Figure 3). Filling level, number of disposals, firmware version, signal strength, and network operator are some of the collected parameters. On the other hand, if the cloud link is unavailable the smart bin locally stores relevant disposal information, delivering it via vehicular fog upon the next oil collection (see Figure 4).

The smart bin is deployed with a unique QR code, scannable via a mobile application, intended to improve the end-user’s experience and for the user to be able to authenticate its disposals.

### 3.3. SWAN Improvements

By implementing edge computing techniques, this proposed architecture is able to cope with the tasks performed by the smart bin, guaranteeing tangible benefits when comparing to state-of-the-art cloud computing approach:

1. Service Availability
  The introduction of user engagement techniques implies that a smart collection bin is able to, directly or indirectly, exchange data with the cloud in order for every user to be able to benefit from the oil collection campaigns and gamification programs. Context-awareness was also introduced in the edge devices. The smart oil bin periodically checks its network status, falling into one of the behaviors specified in Figure 3 and Figure 4.
  Also, regarding service availability, the end-nodes are currently provided with multimode wide area network communication modules as well as the possibility of national/international roaming between network providers. This feature ensures that, for the vast majority of deployment scenarios, the cloud layer is directly available from the edge nodes’ standpoint.
2. Resource Efficiency
  Ensuring an intelligent use of energy and operating costs makes the difference between a successful or unsuccessful solution. The application of pay-per-use data plans means that there is an associated cost with every communication event. Meanwhile, the energy consumption also significantly increases during communication (cf. Section 4). In order to mitigate energy consumption and total cost of operation, a dynamic communication paradigm is proposed. Aspects like current filling level, variance in the filling level, and time elapsed since the previous communication event are evaluated prior to any potential data exchange.
3. Network Offloading
  As the number of deployed units increases, the load over the used, resource-constrained, wide area networks grows, although the data sent to the cloud on the current iteration consist of low-volume telemetry. Current developments are assessing the possibility of oil classification upon each disposal [35]. Since this complex task relies on computer vision techniques to gather inputs for classification, in a cloud computing approach, the data exchange between the edge and cloud layers tends to increase, overloading the communication link. In an edge computing approach, SWAN proposes a place in which data collection and classification is performed. In Figure 3 and Figure 4, it is specified that prior to sending any telemetry, the edge device needs to process its current local state, which means that only preprocessed/classified data are sent to the cloud/fog layer.
4. Security And Privacy
  In a scenario where the smart cooking oil collection bin collects sensitive data upon each disposal, security mechanisms should be implemented in order to cope with the strictest data protection rules (e.g., European General Data Protection Rules—GDPR [34]). Given the resource-constrained nature of IoT devices, the smart collection bin cannot rely solely on complex encryption techniques to ensure privacy and data security [38]. Therefore, besides implementing the state of the art standards on IoT security, SWAN proposes an architecture-level second layer of security by specifying two distinct communication channels. A low-bandwidth, long-range channel is aimed exclusively at nonsensitive telemetry (e.g., filling level) and a secure-oriented, second one (via vehicular fog) is used for the exchange of user data and system-related configurations (see Figure 2).

With the aim of tackling the challenges raised by a widely spread network of smart oil bins, SWAN proposes a solution for an efficient oil collection task. Service availability, operation costs, energy consumption, and the load over the network are some of the benefits brought about by SWAN. We believe that this approach may improve the perception of final users, public entities, and management operators and, therefore, its chances of success.

## 4. Evaluation

Over a period of four years, data were collected from the presented UCO collection network. The data analyzed included the location and number of publicly available bins, oil collections, and on-field technical interventions. This analysis was conducted to evaluate both user engagement and the efficiency of the collection process.

### 4.1. User Engagement

**Figure 5:**
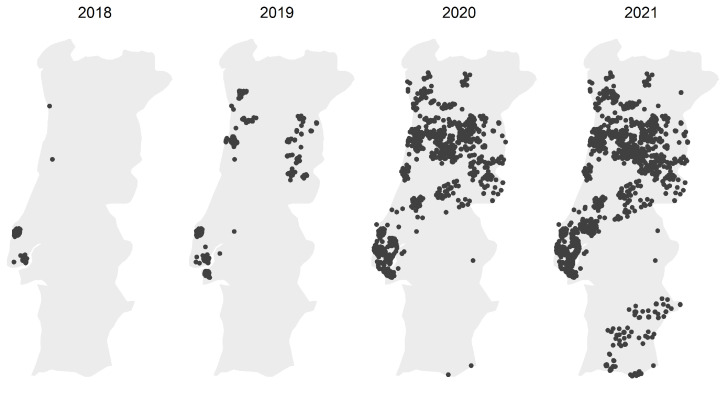
Installed UCO collection bins from January 2018 to December 2021.

Before increasing the number of active users over the installed network, the focus was kept on ensuring that the smart cooking oil collection bin was a commercially interesting product, easing the job of deploying a vast network of units. Figure 5 displays the evolution of the cumulative number of active collection bins in the Portuguese UCO collection network.

With a countrywide network of smart collection bins that citizens could rely on, the monthly totals of collected UCO saw an exponential increase over the years. A more comprehensive coverage, along with the application of user engagement techniques supported by the smart bin, contributed to the fact that, by the end of 2021, the total UCO collected by Hardlevel already exceeded the national totals registered in the years prior to the system implementation.

Although the increase in collected oil serves as a good indicator of the system’s performance, the assessment of user engagement lacks a comparative analysis between the accumulated sum of installed oil bins and the total oil collected. In fact, in line with the growth in oil collections, the number of installed oil bins has maintained an increasing trend. However, looking at the network evolution presented in Figure 5, the majority of new installations is tending towards regions with lower population density where fewer collections are expected.

**Figure 6:**
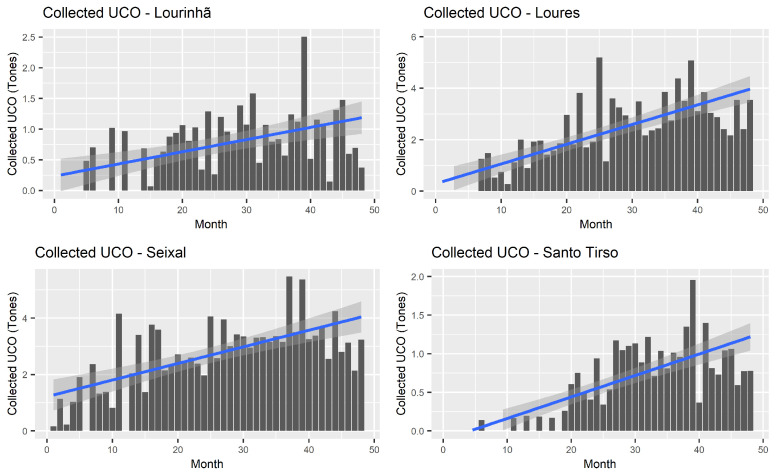
Monthly totals of UCO in a sample of four selected regions from January 2018 to December 2021 including a trend line wthin a confidence interval.

To achieve a representative sample, four regions with different collection volumes were selected from the total ones installed between 2018 and 2019 in the first deployments. Although in different magnitudes, the quantities collected registered a positive evolution, with some regions more than doubling the collected quantities. Figure 6 exhibits the monthly evolution of the four distinct regions: Lourinhã, Loures, Seixal, and Santo Tirso.

### 4.2. Edge Device Consumption and Oil Collection Efficiency

A major goal of the smart oil bin is to maximize the collected oil efficiency upon each visit to the UCO collection bin. This means that, ideally, the oil management operator team should visit the UCO bin only when it is nearly full or requires service maintenance operation. Although no real comparative data are yet available to compare efficiency differences between a standard and a smart cooking oil collection unit, it can be easily accepted that a standard, suboptimal approach of oil collection based on predefined and fixed intervals leads to an overall lower efficiency when compared to a smart “as needed” collection and visiting policy.

In addition to collections, battery replacements and technical interventions are some of the reasons that justify a visit to the smart oil bin. Since energy consumption is highly dependent on the chosen hardware and software solutions, improvements in the collection efficiency are only possible with a smart power usage during both sleep and active/awake periods.

**Figure 7:**
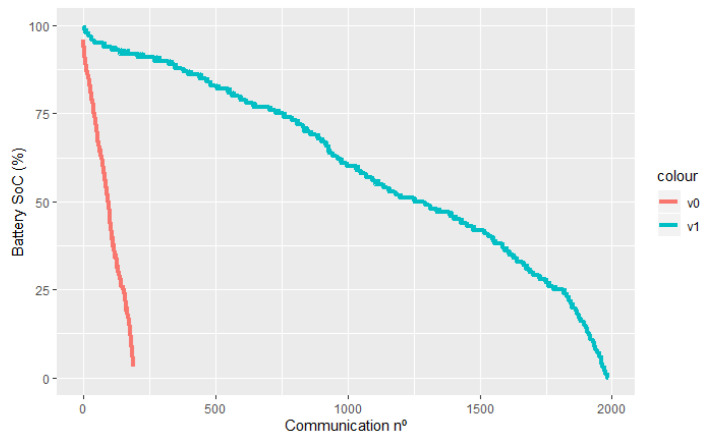
Battery discharge curves over number of communications for both software versions (*v0*: non-SWAN edge device; *v1*: SWAN edge device).

Figure 7 evaluates the influence of software improvements over time/energy spent in each communication, while Table 3 displays the evolution of the applied hardware regarding its quiescent electric current ($I_{sleep}$), extrapolating a theoretical battery life (in days) for an ever-sleeping node.

It is worth noting that, although real-world data are available for the first iterations, the lack of comprehensive results for the latest hardware and software solutions justifies the exclusive usage of in-lab values, minimizing the variables that can affect the comparison.

A node completes a duty cycle with the combination of a sleep and active period, which means that, for estimating the battery life in a production environment, it is necessary to cross both results of Table 3 and Figure 7. For instance, assuming a node communicates once a day and keeps a sleep state for the remaining time, the expected battery life could be estimated as follows:

Average current ($I_{avg}$) during a complete duty cycle of one day (1 day = 86,400 s) is:

$$I_{avg}=\frac{T_{awake}}{86,\;400}I_{awake}+\frac{T_{sleep}}{86,\;400}I_{sleep}$$

with $T_{awake}$ and $T_{sleep}$ expressed in seconds and $T_{sleep}=86,\;400-T_{awake}$. Given an electric current consumption of $I_{avg}$ in (mA) and a fully charged battery energy capacity $E_{battery}$ in (mAh), the battery life ($T_{battery}$), in hours, can be easily calculated as follows [26]:

$$E_{battery}=I_{avg}T_{battery}\Leftrightarrow T_{battery}=\frac{E_{battery}}{I_{avg}}$$

Table 4 presents the expected battery life of the two HW versions. Communication on a daily basis and a battery of 3100 mAh were considered.

When we directly compare the two iterations in a deployment-like environment, the implemented hardware and software updates justify the battery life increase of nearly ten times. Even considering variables like battery self-discharging or extreme operating temperatures [39], the overscaled energy plant should ensure that a visit to a collection unit does not happen to (exclusively) replace batteries, with exchanges taking place simultaneously with oil collections.

In summary, the obtained results for both user engagement and collection efficiency were positive. The focus on making the smart cooking oil collection bin a commercially viable product facilitated a swift expansion in the number of installed units. The user engagement techniques implemented played a significant role in the increase in oil collection per bin. Moreover, the optimization of both software and hardware significantly reduced the operational overhead of a widely deployed network. The filling-level sensing capabilities combined with a low operational overhead led to an increase in the collection efficiency.

## 5. Conclusions

There is an environmental need to recycle used cooking oil and guarantee that it is not disposed of incorrectly. The lack of awareness-raising actions and the scarcity of widely available collection networks are affecting the percentage of UCO directed towards recycling in the domestic sector. Recognizing that a successful collection network needs to meet the interests of all stakeholders, an IoT-enabled network of collection bins was developed and deployed. The SWAN system proposes a custom edge-computing architecture tailored for providing municipalities with a clean and smart city-blended network of bins. It also involves citizens with an enriched and functional UCO disposal system, managed by a single company fostering optimized collection and management tasks.

Based on four years of accumulated data and an exponential increase in annual collected oil, it is now possible to state that, in the context of domestic used cooking oil collection, SWAN presents an interesting, viable approach capable of disruptively changing the UCO collection landscape.

Although successfully validated, the developed solution is currently evolving. Along with the increase in active users, the number of untrustworthy oil disposals has also increased. Therefore, in order to ensure that only used cooking oil disposals are rewarded, current and future work is targeting the use of machine learning classifiers to detect untrustworthy oil disposals. This edge AI-enabled solution will integrate directly within SWAN, increasing all its known benefits.

### Future Work

Looking ahead, there are several areas where future research could enhance the SWAN system. As our dataset grows and the SWAN system matures, the use of machine learning techniques offers the potential to optimize waste collection process even more by analyzing the vast amounts of data to predict optimal collection routes, reduce operational costs, and minimize environmental impact. Beyond the ongoing work on machine learning classifiers, other potential areas of focus include the expansion of the SWAN system to other types of waste collection. The principles and technologies used in SWAN could potentially be adapted to manage other forms of household waste, contributing to more comprehensive and efficient waste management systems.

Moreover, future work could also explore ways to further engage and incentivize citizens. This could include developing more interactive features, or implementing reward systems that provide greater benefits to consistent and responsible users.

By providing the specification and assessment of a full stack collection network, we encourage this work to be applied in other application scenarios. We believe that the lessons learned from the development and deployment of SWAN can provide valuable insights for researchers and practitioners working on similar challenges in waste management in smart city applications.

## Figures and Tables

**Table 1:** Cloud vs. edge vs. fog computing.

Criteria	Cloud	Edge	Fog
Architecture	centralized	decentralized	decentralized
Security	low	high	high
Energy consumption	low	high	average
Location awareness	✗	✓	✓
Mobility	✗	✓	✓
Latency	high	low	medium
Computing and storage	high	low	average
Scalability	average	high	high

**Table 2:** Comparison of related work on IoT-enabled waste management solutions.

Project	User Engagement	Collection Efficiency	Scalability
Sousa et al. [25]	Marginal	Good	Marginal
Gomes et al. [26], Costa et al. [27]	Marginal	Good	Good
Cao et al. [31]	Marginal	Excellent	Good
Iqbal et al. [30]	Marginal	Good	Good

**Table 3:** Quiescent current ($I_{sleep}$) comparison per version (*v0*: non-SWAN edge device; *v1*: SWAN edge device).

Edge HW/SW Version	Quiescent Current (mA)	Theoretical Max (Days)
*v0*	0.48	269
*v1*	0.015	8611

**Table 4:** Expected battery life comparison per version (*v0*: non-SWAN edge device; *v1*: SWAN edge device).

Edge HW/SW Version	$T_{\mathrm{awake}}$ (s)	$I_{\mathrm{awake}}$ (mA)	Expected Battery Life (Days)
*v0*	170	120	179
*v1*	29	180	1713

## Data Availability

The data-sets generated and analyzed during the current study are available from the corresponding author on reasonable request.
